# Exploring the potentials of halophilic prokaryotes from a solar saltern for synthesizing nanoparticles: The case of silver and selenium

**DOI:** 10.1371/journal.pone.0229886

**Published:** 2020-03-04

**Authors:** Maryam Abdollahnia, Ali Makhdoumi, Mansour Mashreghi, Hossein Eshghi

**Affiliations:** 1 Department of Biology, Faculty of Science, Ferdowsi University of Mashhad, Mashhad, Iran; 2 Center of Nano Research, Ferdowsi University of Mashhad, Mashhad, Iran; 3 Department of Chemistry, Faculty of Science, Ferdowsi University of Mashhad, Mashhad, Iran; Karl-Franzens-Universitat Graz, AUSTRIA

## Abstract

Halophiles are the organisms that thrive in extreme high salt environments. Despite the extensive studies on their biotechnological potentials, the ability of halophilic prokaryotes for the synthesis of nanoparticles has remained understudied. In this study, the archaeal and bacterial halophiles from a solar saltern were investigated for the intracellular/extracellular synthesis of silver and selenium nanoparticles. Silver nanoparticles were produced by the archaeal *Haloferax* sp. (AgNP-A, intracellular) and the bacterial *Halomonas* sp. (AgNP-B, extracellular), while the intracellular selenium nanoparticles were produced by the archaeal *Halogeometricum* sp. (SeNP-A) and the bacterial *Bacillus* sp. (SeNP-B). The nanoparticles were characterized by various techniques including UV-Vis spectroscopy, XRD, DLS, ICP-OES, Zeta potentials, FTIR, EDX, SEM, and TEM. The average particle size of AgNP-A and AgNP-B was 26.34 nm and 22 nm based on TEM analysis. Also, the characteristic Bragg peaks of face-centered cubic with crystallite domain sizes of 13.01 nm and 6.13 nm were observed in XRD analysis, respectively. Crystallographic characterization of SeNP-A and SeNP-B strains showed a hexagonal crystallite structure with domain sizes of 30.63 nm and 29.48 nm and average sizes of 111.6 nm and 141.6 nm according to TEM analysis, respectively. The polydispersity index of AgNP-A, AgNP-B, SeNP-A, and SeNP-B was determined as 0.26, 0.28, 0.27, and 0.36 and revealed high uniformity of the nanoparticles. All of the synthesized nanoparticles were stable and their zeta potentials were calculated as (mV): ˗33.12, ˗35.9, ˗31.2, and ˗29.34 for AgNP-A, AgNP-B, SeNP-A, and SeNP-B, respectively. The nanoparticles showed the antibacterial activity against various bacterial pathogens. The results of this study suggested that the (extremely) halophilic prokaryotes have great potentials for the green synthesis of nanoparticles.

## Introduction

Halophilic prokaryotes are salt-loving microorganisms that cannot survive in the absence of salts (most special NaCl). They are classified into moderate and extreme halophiles based on the amounts of salt that support their best growth. Moderate halophiles grow optimally under 1.5 M salt concentrations, and most of them belong to the *Bacteria*. They use some organic compounds called compatible solutes (compatible with cellular macromolecules) to counter the high osmosis pressure. On the other hand, extreme halophiles grow optimally in the presence of more than 1.5 M salts (up to 5 M), and most of them belong to the *Archaea*. They store a high amount of salts (especially KCl) inside their cells to balance the osmosis pressure [1]. Halophiles could be obtained generally from salt concentered ecosystems in the world, i.e. salt lakes where the high rate of evaporation has increased the salinity of water by 5–10 times more than marine environments (30% salinity). Solar salterns are artificial saline lakes in tropical and sub-tropical regions which consist of a series of ponds filled with seawater. The salinity increases gradually through the effects of the wind and temperature with the salt produced at the last pond being called the crystallizer pond [2]. Living in these extreme environments (high salt concentration, high UV radiation, low water content) requires unique cell components, different form non-halophile (micro)organisms. Consequently, halophile macromolecules have the potential to be used in biotechnological processes such as biopolymers, hydrolytic enzymes, biosurfactants, biofuels, and bioremediation which are not suitable for common prokaryotes [3].

Nanotechnology is an emerging science which considers the elements in their nano-scales (1–1000 nm). Compared to their bulk counterparts, nanomaterials offer unique features such as a high surface to volume ratio and quantum effects allowing the tunability of some of their physicochemical properties [4]. There are growing applications of nanomaterials in the various industrial sectors including (but not limited to) food, electronics, pharmaceuticals, fuels, chemicals, polymers, and environmental health [5–6]. Conversion of compounds from the bulk (regular) scale to the nano-size is the first step for the application of nanomaterials in nanotechnology. Various physicochemical (top-down) approaches have been presented for the synthesis of nanomaterials. However, these methods are relatively expensive, have high propensity of agglomeration, and some hazardous chemicals for the environments are used/produced [7]. Bio-based approaches which employ live organisms or their products for synthesizing nanoparticles have gained interest due to their high efficiency and compatibility with the environment (green approach). Nanoparticles produced by biogenic approaches have greater stability with respect to the cellular stabilizing capping metabolites [8].

Halophilic prokaryotes can tolerate and detoxify heavy metals by various mechanisms like extracellular metal sequestration or intracellular enzymatic reduction [9]. During these detoxification processes metallic ions could convert to nanoparticles. Although extensive studies have been performed on the remediation applications of halophiles, the abilities of these extremophiles for the synthesis of nanoparticles have remained understudied (especially for haloarchaea), and only a few reports are available in this context [10]. In the current study, the halophilic prokaryotes (moderate and extreme) were isolated from a solar saltern pond, and their potentials were investigated for the green synthesis of two broadly used nanoparticles, i.e., silver and selenium. The characteristics of the obtained nanoparticles were evaluated in detail and compared with previously reported nanoparticles with an emphasis on the role of salt.

## Materials and methods

### Site description, prokaryotes isolation, and identification

Samples were taken from the crystallizer ponds of the Tis solar saltern in the southeast of Iran by the shore of Oman gulf (25°22′19′′ N, 60°36′46′′ E). All necessary permits were obtained from the local Department of Environment for the described field studies. Halophilic prokaryotes were isolated under aerobic conditions on a Salt Water (SW) medium consisting of (g/l) peptone 1.0, yeast extract 0.2 and agar 15.0, pH 7.2; containing 10% and 20% (W/V) of its total salts (NaCl 156, MgCl_2_.6H_2_O 27.7, MgSO_4_.7H_2_O 39.6, CaCl_2_ 0.76, KCl 4, NaHCO_3_ 0.2, NaBr 0.5) [11]. The plates were incubated aerobically at 40°C for four weeks. After successive cultivation, pure isolates were obtained. The requirements and optimum amounts of NaCl for the growth were determined in the broth medium containing 0–5 M NaCl. Anisomycin (Santa Cruz, USA) susceptibility test was carried out according to the disk diffusion method at a concentration of 30 μg per disk to distinguish between bacterial and archaeal strains [12]. The extraction of genomic DNA, PCR amplification of the 16S rRNA gene, and phylogenetic analysis of the selected strains were carried out as described elsewhere [13].

### Screening for resistance of heavy metals in halophile strains

Minimum inhibitory concentration (MIC) of silver and selenium salts was determined in SW broth medium. The medium was supplemented with different silver nitrate and sodium selenite concentrations ranging from 0.05 to 1 mM (0.05 intervals) and 0.5 to 5 mM (0.5 intervals), respectively. The growth was determined by measuring the absorbance at 600 nm after the cultivation at 40°C in the dark under non-agitated conditions and compared with the two control mediums (without metals and non-inoculated).

### Biosynthesis of nanoparticles

#### Intracellular synthesis of nanoparticles

Bacterial (moderate halophile) and archaeal strains (extreme halophile) were inoculated in 1000 ml Erlenmeyer flasks containing 200 ml 10% and 20% SW broth medium supplemented with filter-sterilized AgNO_3_ (0.5mM) and Na_2_SeO_3_ (5mM). The flasks were incubated in an orbital shaker at 40°C and agitated at 150 rpm for 3 and 7 days, for the bacterial and archaeal strains, respectively. The un-inoculated 10% and 20% SW broth media supplemented with equal concentrations of silver nitrate and sodium selenite were incubated simultaneously as control.

#### Extracellular synthesis of nanoparticles

Bacterial (moderate halophile) and archaeal strains (extreme halophile) were inoculated in 1000 ml Erlenmeyer flasks containing 200 ml 10% and 20% SW broth medium, respectively. The flasks were incubated in an orbital shaker at 40°C and agitated at 150 rpm for 3 and 7 days, for the bacterial and archaeal strains, respectively. The culture supernatant was obtained by centrifugation at 10000 rpm for 15 min, filtered through a 0.22 μm syringe filter and used for the synthesis of the nanoparticles. For the extracellular production of nanoparticles, the filter-sterilized AgNO_3_ and Na_2_SeO_3_ were added to the cell-free supernatant (CFS) with the final concentrations of (mM) 0.5 and 5.0, respectively. The mixtures were then autoclaved at 121°C under 15 psi pressure for 20 min. The un-inoculated 10% and 20% SW broth media supplemented with equal concentrations of silver nitrate and sodium selenite were used as the control [14]. A part of the cell-free supernatant (CFS) was freeze-dried (Freeze dryer, Christ Alpha 1.2 LD Plus, Germany) and applied for FTIR analysis.

### Purification of the nanoparticles

#### Intracellular NPs

For the recovery of the intracellular nanoparticles, both archaeal and bacterial cells were disrupted. The microbial biomasses were collected through centrifugation of the culture media at 10000 rpm for 20 min. Archaeal cells were simply lysed in distilled water. In order to destroy the bacterial cells, the washed cells were resuspended in the lysis buffer (lysozyme 1% (w/v) and SDS 1% (w/v)) and incubated at 37°C for 5 h at two intervals of sonication (15 min). The cell lysates were mixed with the half volume of 1-octanol, mixed thoroughly and centrifuged at 10000 rpm for 20 min, then kept for 24 h at 4°C. The supernatant was discarded carefully and sediment nanoparticles were washed accordingly in chloroform, absolute ethanol, 70% (v/v) ethanol and deionized water. Finally, the nanoparticles were dispersed in a small amount of deionized water using ultrasonic bath and dried in a vacuum oven at 37°C for 24 h [15].

#### Extracellular NPs

Nanoparticles produced by CFS were collected through centrifuging the reaction mixtures at 10000 rpm for 30 min. The obtained nanoparticles were washed three times with deionized water and then successively rinsed in the absolute ethanol, 70% (v/v) ethanol and deionized water. Finally, they were dispersed in a small amount of deionized water using ultrasonic bath and dried in the vacuum oven at 37°C for 24 h.

### Characterization of nanoparticles

#### UV-visible spectroscopy

The formation of AgNPs and SeNPs was confirmed preliminary through visual observation of color changes in the supernatants/culture media. The optical absorbance of the nanoparticles dispersed in distilled water was detected on a Unico UV-2100 spectrophotometer (Agilent, Palo Alto, CA, USA) at a resolution of 5 nm from 200–800 nm with distilled water as the blank. The UV–visible spectra of the sodium selenite and silver nitrate solutions were also recorded as controls.

#### X-ray diffraction (XRD)

Crystallographic characterization of the purified nanoparticles was performed by Bragg-Brentano geometry using D8-Advance diffractometer (Bruker, Germany). The XRD patterns were recorded at 40 kV/40 mA with Cu-Kα1 (1.54 A^o^) as a radiation source within the range 10° ≤ 2θ ≤80° with a step size of 0.04° per second. The results of XRD patterns were analyzed through Match! Software version 3.3.0. The crystallite sizes were calculated using Debye Scherrer’s equation:

D=Kλβcosθ (1)

Where, D is the crystallite size, K represents a constant taken to be 0.94, λ reflects the wavelength of the x-ray radiation, β denotes the full width at half maximum (FWHM), and θ is the angle of diffraction [16].

#### Inductively coupled plasma—Optical emission spectrometry (ICP-OES)

The efficiency of the production of nanoparticles was determined based on the measurements of the remaining amount of primary silver and selenite salts in the culture media/reaction mixtures by the inductively coupled plasma optical emission spectrometry (ICP-OES) (SPECTRO ARCOS, Germany) [17].

#### Zeta potential

The surface charge of the nanoparticles was quantified as zeta potential (ζ). It was measured based on micro-electrophoresis technique by a ZetaCompact laser zeta meter (CAD, Les Essarts-le-Roi, France) at 25°C, and pH 6.17 ± 0.1 for AgNPs and pH 6.53 ± 0.1 for SeNPs, respectively [18].

#### Dynamic Light Scattering (DLS)

The particle size distributions of the biosynthesized nanoparticles were determined using a VASCO-3 nanoparticle size analyzer (Cordouan Technologies, Pessac, France) after 30 min homogenization with ultrasound bath at 25°C with cumulants method. Measurements were taken within the range from 0.5 up to 10000 nm [19].

#### Fourier transform infrared spectroscopy (FT-IR)

In order to identify the possible functional groups in the surface of nanoparticles and filtrated cell free supernatant (CFS), FT-IR spectra were analyzed using the KBr pellet method within the range of 400–4000 cm^-1^ at a resolution of 4 cm^-1^ by AVATAR 370 FT-IR spectrophotometer (Madison, USA) [20].

#### Atomic force microscopy (AFM)

Surface topography and size of the nanoparticles were characterized by a multimode full plus model AFM 0101/A instrument (Ara Research, Iran). An aliquot of dispersed nanoparticles was spread evenly on to a freshly cleaved mica sheet and then dried at ambient temperature. AFM images were obtained in the tapping mode using a silicon nanoprobe cantilever with the tip height 10 nm and the resonance frequency of 218 kHz. Two-dimensional images were used to estimate the average particle size using the instrument software (Imager version1.01). Three-dimensional topographic images were used to generate the mean particle height [21].

#### Transmission and Scanning electron microscopy (TEM and SEM)

The morphological characteristics of intracellular NPs as well as their interactions with microbial cells, were examined using SEM. Microbial cells were harvested from culture media and washed three times, with 15% salt water. The pellets were fixed for 2 h in 2.5% glutaraldehyde at room temperature, dehydrated, and then coated with gold [22]. The samples were observed under LEO 1450 VP- Zeiss scanning electron microscope (SEM) (Jena, Germany) at the resolution of 2.5 nm and an accelerating voltage of 20 kV. Energy-Dispersive X-ray (EDX) analyses were carried out simultaneously to determine the elemental composition of nanoparticles by INCAx-act Oxford Instruments at the resolution of 133 ev (High Wycombe, UK). All synthesized nanoparticles (extra/intracellular approaches) were dispersed in deionized water via sonication (Fungilab ultrasonic cleaner) at 0°C for 30 minutes each at 2 RPS (160 W). The obtained colloidal solutions were dropped on carbon-coated copper TEM grids, followed by drying at ambient temperature for 24 hours. The morphological characteristics of the nanoparticles were analyzed on a Leo 912 AB TEM instrument (Zeiss, Germany) at an accelerating voltage of 120 kV at different magnifications. The particle size distribution was calculated by measuring the diameters of 100 NPs through Smile View 2.0 software (Tokyo, Japan).

#### Antibacterial activity

The minimum inhibitory concentration (MIC) of the biologically‏ synthesized NPs against four reference strains including *Escherichia coli* ATTCC 8739, *Pseudomonas aeroginosa* ATCC 9024, *Staphylococcus aureus* ATCC 6538 and *Bacillus subtilis* ATCC 6051 was determined using‏ the broth microdilution method [23].

### Statistical analysis

All quantitative experiments were carried out as three independent experiments. The standard error of the mean was calculated and results shown in the figures and text represent the mean *±* standard deviation. One-way analysis of variance was done through SPSS ver. 16 software (IBM Co) for the antibacterial assay.

## Results

### Selection of prokaryotes with the ability to produce nanoparticles

A total of 100 prokaryotic strains were obtained from the Tis solar saltern. Of them, 59 strains were extreme halophiles, 31 strains were moderate halophiles, and the remaining nine strains were halotolerant. All the extreme halophiles were identified as *Archaea* and all the moderates were identified as *Bacteria* based on their susceptibility and resistance to the anisomycin, respectively. A total of 24 and 10 strains of archaeal and bacterial strains could grow up to 1.0 mM AgNO_3_, respectively and selected for the synthesis experiments. Regarding the Na_2_SeO_3_, a total of 31 and 26 strains belonging to the archaeal and bacterial strains could survive up to 5.0 mM salt concentration and selected for further experiments. S1 Fig. represents the resistance patterns of isolated strains to the silver nitrate and sodium selenite salts.

### AgNPs synthesis

**Fig 1:**
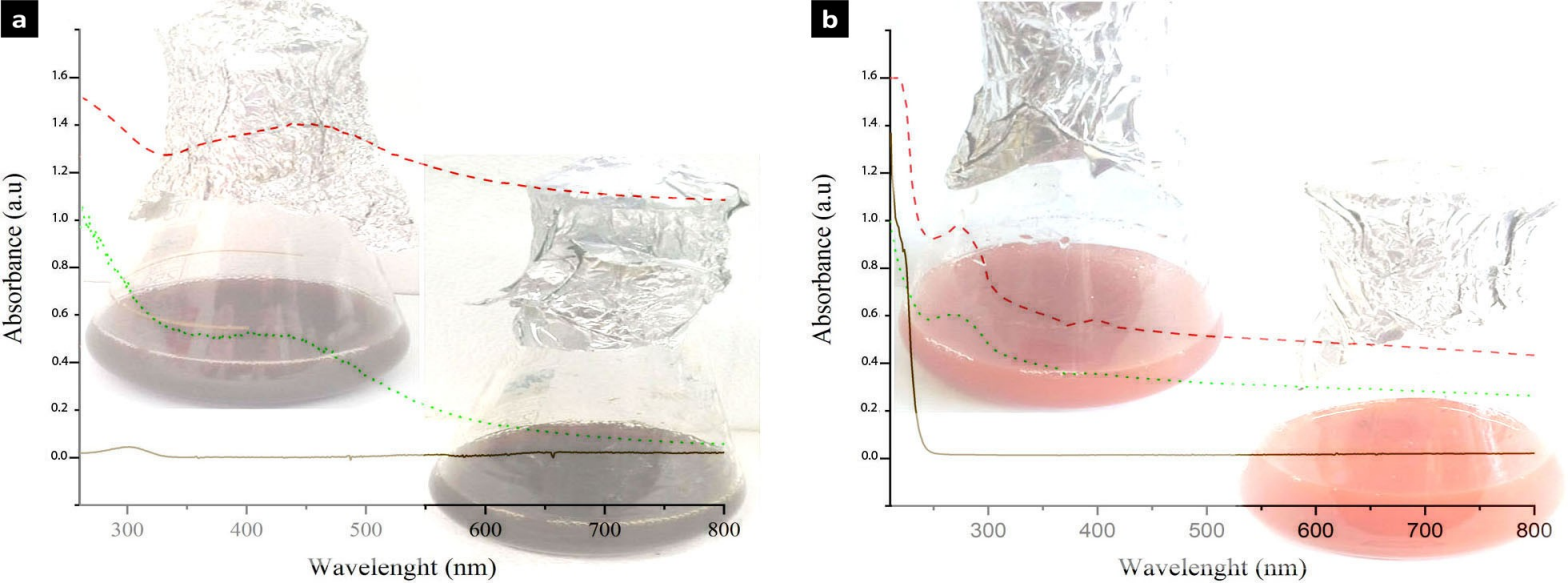
UV-visible absorbance of the silver (a) and selenium (b) nanoparticles. Dash: archaea, dot: bacteria, solid: silver and selenium salts. Inlet: reduction of silver and selenium salts evidenced by color change of reaction mixture from to dark brown and red, respectively (up: archaea, down: bacteria).

The ability of the halophilic archaea and bacteria was determined for the synthesis of silver nanoparticles by both intracellular and extracellular approaches. The appearance of a dark brown color in the culture media/CFS signifies the probable formation of nanoparticles (Fig 1A inlet, up). Among the archaeal strains, strain E106 was the most potent (visual detection, S2 Fig) and could produce AgNPs in both intracellular and extracellular approaches. However, the intracellular approach for the archaeal synthesis of the silver nanoparticles, based on the properties of produced nanoparticles, was selected (we discuss it later). Regarding the bacterial strains, none of them could produce AgNPs by the intracellular method, and only the CFS (extracellular approach) of the strain D1 could produce nanoparticles and was selected for the synthesis procedure (Fig 1A inlet, down).

### SeNPs synthesis

All selected archaeal and bacterial strains could produce SeNPs by both intracellular and extracellular approaches (based on the preliminary appearance of a brick-red color in the culture media (Fig 1B inlet, up: archaea, down: bacteria). According to the degrees of NPs production (visual detection, S1 Fig), strains E118 (archaea) and D54 (bacteria) were selected for the intracellular synthesis of the selenium nanoparticles in further analysis.

In the following sections, the characteristics of four nanoparticles are presented. For simplicity, four nanoparticles were coded as AgNP-A (silver nanoparticles synthesized by archaeal strain E106), AgNP-B (silver nanoparticles synthesized by bacterial strain D1), SeNP-A (selenium nanoparticles synthesized by archaeal strain E118), and SeNP-B (selenium nanoparticles synthesized by bacterial strain D54). In these four category designations, only AgNP-B was produced by CFS (extracellular approach) while the other three nanoparticles (AgNP-A, SeNP-A, SeNP-B) were produced intracellulary.

### Identification of the halophilic strains

The four prokaryote strains used for the synthesis of nanoparticle were designated as strains E106 (AgNP-A), D1 (AgNP-B), E118 (SeNP-A), and D54 (SeNP-B). Analysis of the *16S rRNA* gene sequence of strains E 106 (Gene Bank accession number: MN750237), D1 (MN750224), E118 (MN750238), and D54 (MN750231) showed that the obtained strains are members of the *Haloferax*, *Halomonas*, *Halogeometricum* and *Bacillus*, respectively. The closest identified taxa to strains E106, D1, E118, and D54 were *Haloferax denitrificans*, *Halomonas koreensis*, *Halogeometricum borinquense*, and *Bacillus oceanisediminis* with 99.6, 98.5, 99.9, and 99.4% sequence similarity, respectively.

### Characterization of nanoparticles

#### UV–visible spectroscopy

The formations of the nanoparticles were confirmed by UV spectral, XRD, and ICP analyses. The UV–visible spectroscopy results indicated absorption peaks equal to 446 and 425 nm for the purified AgNP-A and AgNP-B, respectively (Fig 1A, up: archaea, down: bacteria). The SeNP-A and SeNP-B revealed the absorption peaks at 275 nm and 270 nm, respectively (Fig 1B, up: archaea, down: bacteria). However, the suspension of silver and selenium salts (AgNO_3_ and Na_2_SeO_3_) did not show any significant peak.

#### XRD

**Fig 2:**
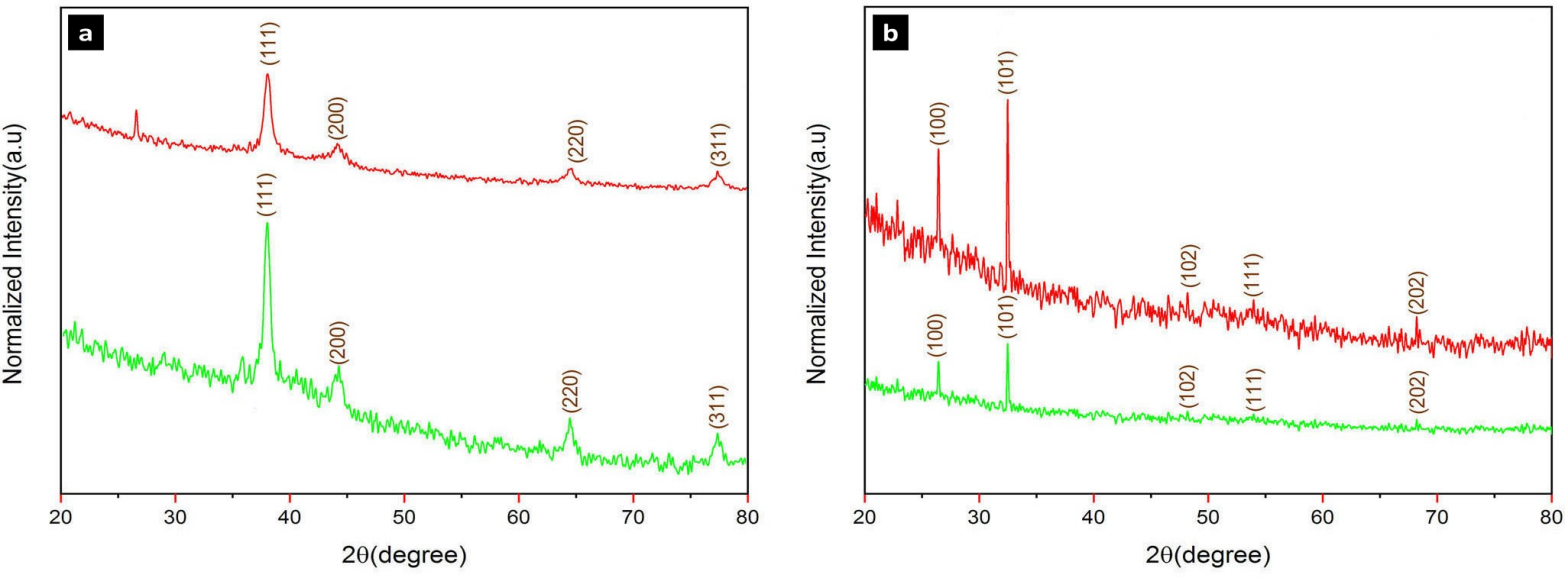
X-ray diffraction (XRD) spectrum of the silver (a) and selenium (b) nanoparticles produced by archaeal (up) and bacterial (down) halophiles.

The X-ray diffraction spectrum of silver and selenium nanoparticles showed intense peaks throughout the entire spectrum 2θ ranging from 20 to 80 as presented in Fig 2. The Bragg’s peaks of 38.2°, 44.4°, 64.6°, and 77.5° corresponded to (111), (200), (220), and (311) facets of the crystalline face centered cubic (fcc) structure of silver nanoparticles for both archaeal and bacterial nanoparticles (Fig 2A, up: archaea, down: bacteria). However, AgNP-A spectrum showed an additional peak at 26.8°. Regarding the selenium nanoparticles, the peaks at 2θ values of 27.2°, 32.5°, 45.0°, 47.2°, 61.2°, along with 24.8°, 30.5°, 43.6°, 47.2°, 60.53° for SeNP-A and SeNP-B, respectively corresponded to (100), (101), (102), (111), and (202) sets of lattice planes for the hexagonal structure for both archaeal and bacterial nanoparticles (Fig 2B, up: archaea, down: bacteria). The crystallite domain sizes were calculated using Debye Scherrer’s equation which were equal to 13.01, 6.13, 30.63, and 29.48 nm for AgNP-A, AgNP-B, SeNP-A, and SeNP-B, respectively. The full width at half maximum (FWHM) values of the nanoparticles were consistent with their size and were calculated as 0.011, 0.024, 0.009, and 0.007 rad for AgNP-A, AgNP-B, SeNP-A, and SeNP-B, respectively.

#### ICP-EOS

The silver and selenite salts concentrations remaining in the synthesis mixtures were determined by ICP-OES and used to determine the efficiency of nanoparticle synthesis. The efficiency of synthesis was 97.6, 85.4, 84.8, and 92.4% for AgNP-A, AgNP-B, SeNP-A, and SeNP-B, respectively.

#### DLS

**Fig 3:**
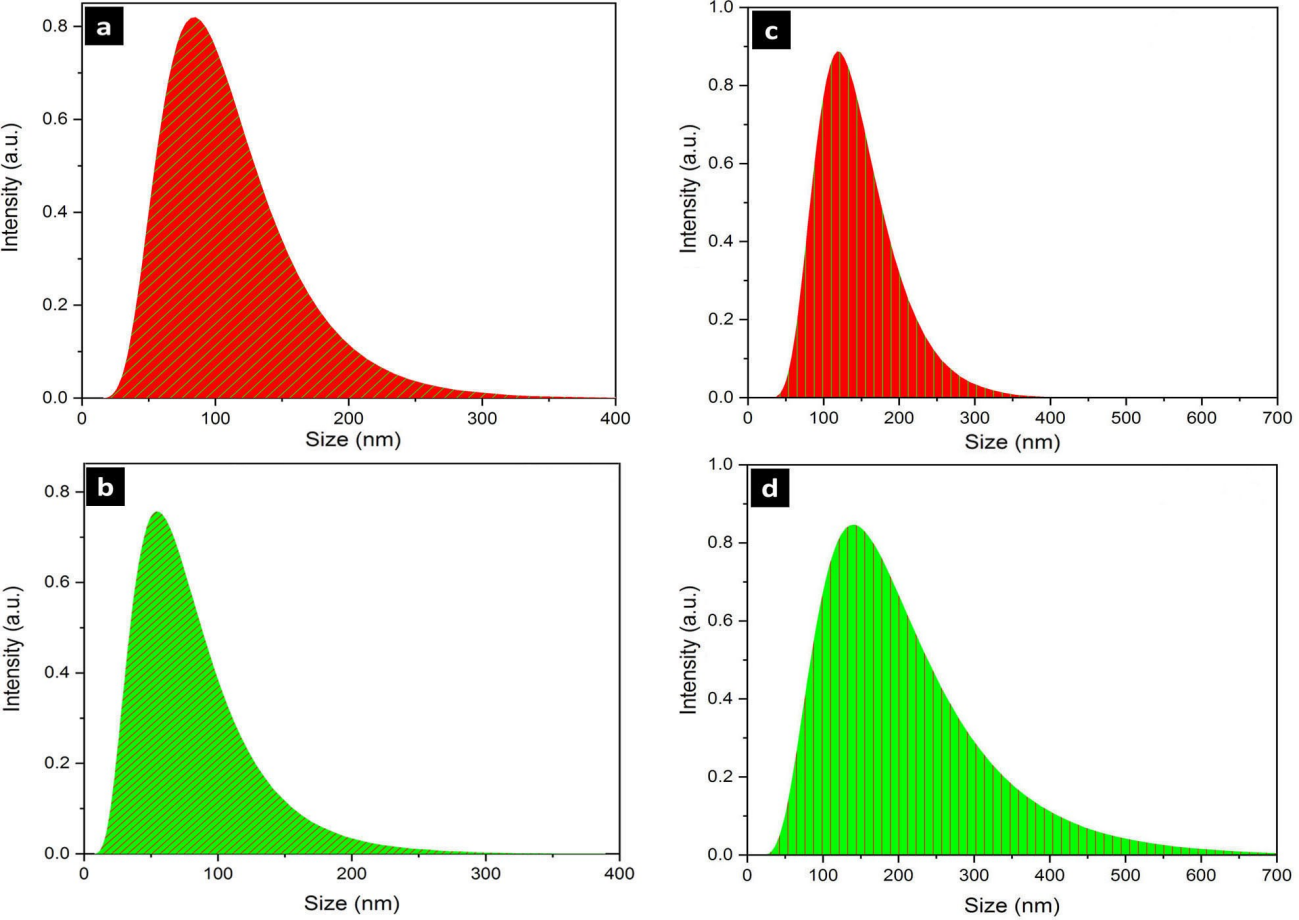
Dynamic light scattering (DLS) of silver and selenium nanoparticles. a, AgNPs-A; b, AgNPs-B; c, SeNPs-A; d, SeNPs-B. AgNPs-A, silver nanoparticles synthesized by archaeal strain; AgNP-B, silver nanoparticles synthesized by bacterial strain; SeNP-A, selenium nanoparticles synthesized by archaeal strain; SeNP-B, selenium nanoparticles synthesized by bacterial strain.

The nanoparticles size and their size distribution were determined by dynamic light scattering analysis (Fig 3A–3D). The silver nanoparticles formed in the archaeal culture (AgNP-A) were within the size range of 16˗213.8 nm with an average particle size of 82.4 nm. The silver nanoparticles produced by bacterial cell free supernatant (AgNP-B) lied within the range of 8.9˗93.3 nm with the average particle size of 52.6 nm. Selenium nanoparticles produced by archaeal (SeNP-A) and bacterial (SeNP-B) cell cultures ranged in size within 29.5˗354.9 nm and 25.7˗708.1 nm, respectively. The average size for the SeNP-A and SeNP-B were as 118.3 nm and 161.9 nm respectively. The polydispersity index (PDI) of AgNP-A, AgNP-B, SeNP-A, and SeNP-B, was determined as 0.26, 0.28, 0.27, and 0.36, respectively.

#### Zeta potentials

**Fig 4:**
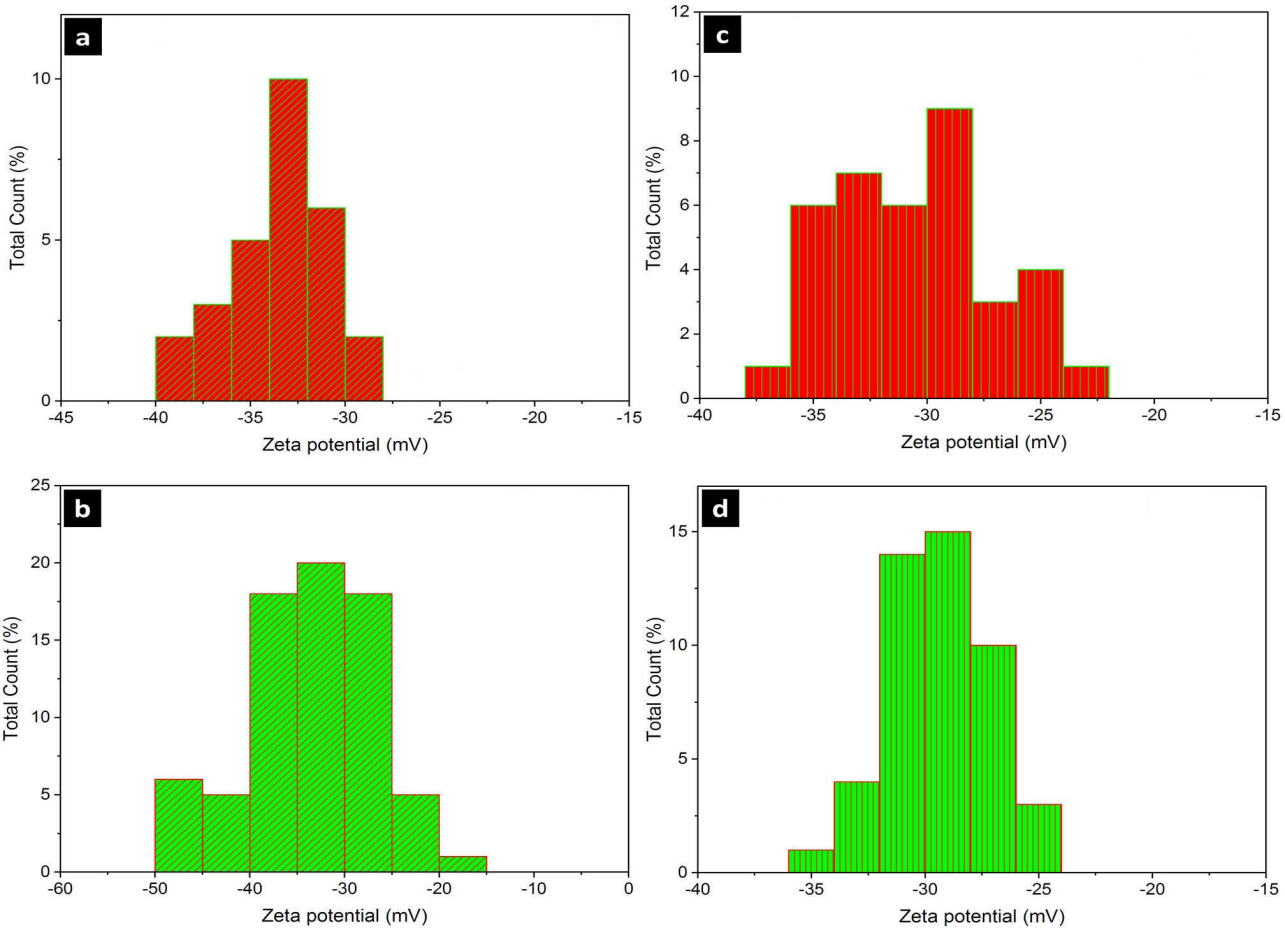
Zeta potential analysis of the silver and selenium nanoparticles. a, AgNPs-A; b, AgNPs-B; c, SeNPs-A; d, SeNPs-B. AgNPs-A, silver nanoparticles synthesized by archaeal strain; AgNP-B, silver nanoparticles synthesized by bacterial strain; SeNP-A, selenium nanoparticles synthesized by archaeal strain; SeNP-B, selenium nanoparticles synthesized by bacterial strain.

The surface charge of nanoparticles in the aqueous solution was measured and presented as Zeta potential. All of the synthesized nanoparticles in this study had a negative surface charge and were calculated as ˗33.12, ˗35.9, ˗31.2, and ˗29.34 (mV) for AgNP-A, AgNP-B, SeNP-A, and SeNP-B, respectively (Fig 4A–4D).

#### AFM

**Fig 5:**
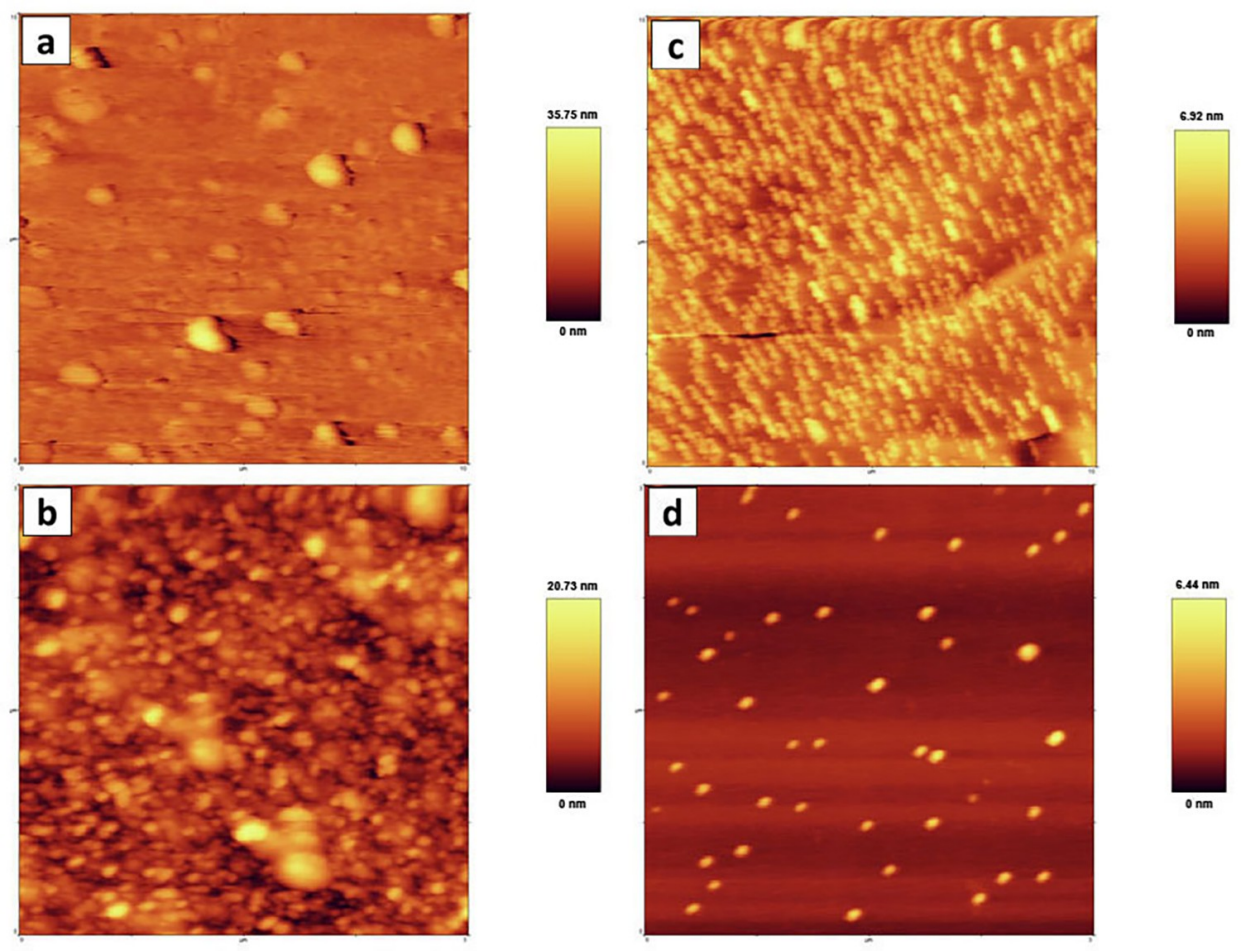
The AFM analysis of the silver and selenium nanoparticles. a, AgNPs-A; b, AgNPs-B; c, SeNPs-A; d, SeNPs-B. AgNPs-A, silver nanoparticles synthesized by archaeal strain; AgNP-B, silver nanoparticles synthesized by bacterial strain; SeNP-A, selenium nanoparticles synthesized by archaeal strain; SeNP-B, selenium nanoparticles synthesized by bacterial strain.

The surface topography, morphology, and size of the obtained nanoparticles are depicted in AFM analysis. Three-dimensional images of nanoparticles revealed that their height were equal to 35.75, 20.73, 6.92, and 6.25 nm for AgNP-A, AgNP-B, SeNP-A, and SeNP-B, respectively. The majority of nanoparticles were symmetrical and spherical in shape; however, some level of agglomeration was observed for the AgNP-B and SeNP-A. The average size measured based on the two-dimensional AFM analysis was 31.6, 24.8, 96.4, and 124.3 nm for the AgNP-A, AgNP-B, SeNP-A, and SeNP-B, respectively (Fig 5A–5D).

#### EDX

**Fig 6:**
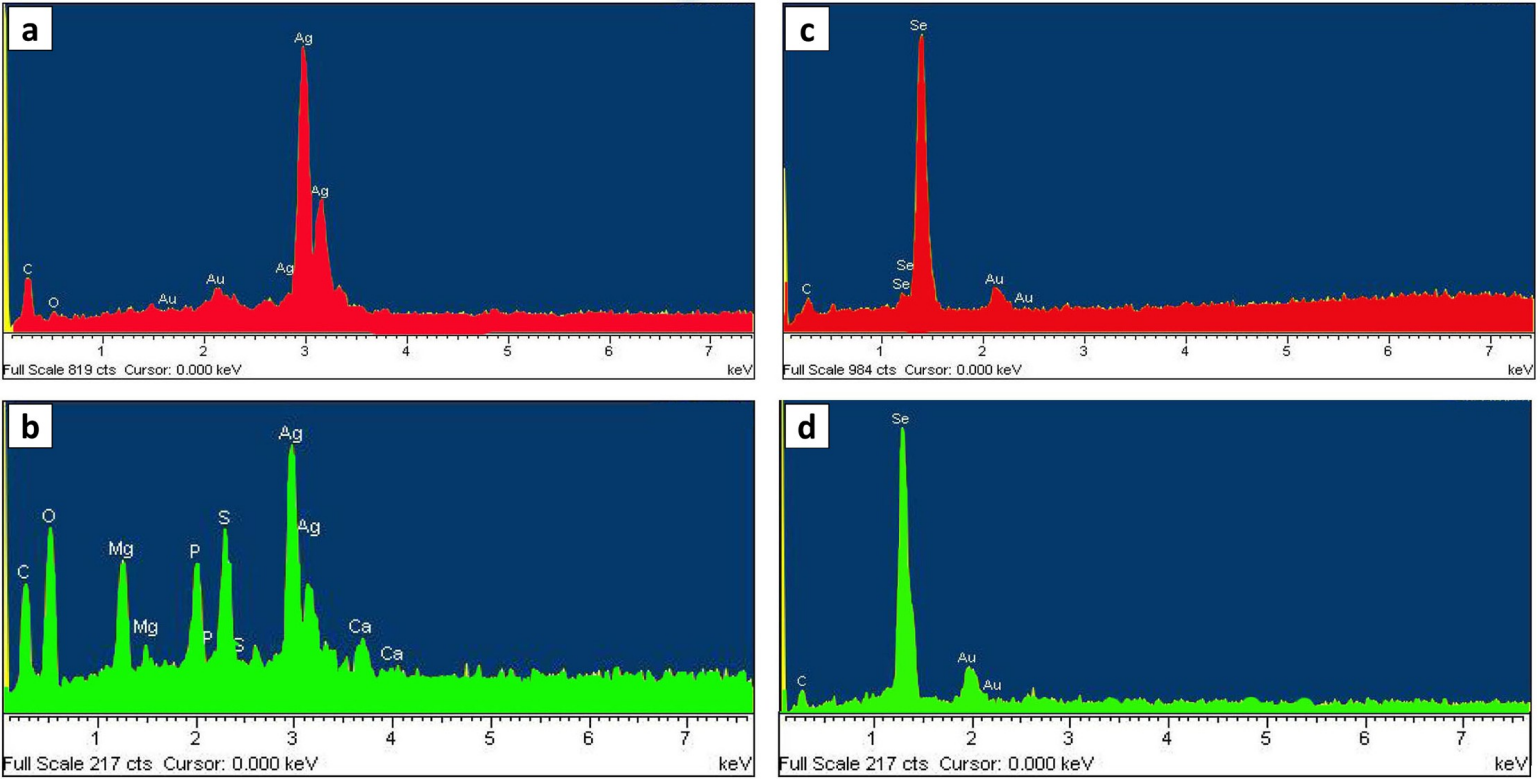
Energy dispersive X-ray (EDX) spectrum of the silver and selenium nanoparticles. a, AgNPs-A; b, AgNPs-B; c, SeNPs-A; d, SeNPs-B. AgNPs-A, silver nanoparticles synthesized by archaeal strain; AgNP-B, silver nanoparticles synthesized by bacterial strain; SeNP-A, selenium nanoparticles synthesized by archaeal strain; SeNP-B, selenium nanoparticles synthesized by bacterial strain.

The elemental composition analysis by EDX showed the presence of strong absorption peaks from Ag (Fig 6A and 6B) and Se (Fig 6C and 6D) atoms at 2.5˗3.5 and 1.0˗2.0 keV, respectively. Additional weak signals of O and C were also observed for all nanoparticles associated with the organic compounds of the samples. Further, some elemental peaks of gold (Au) were also obtained associated with the sample holder and gold coating. However, the AgNP-B which was produced extracellularly by the CFS of strain D1 showed strong noise signals including Ca, S, P and Mg; the salts present in the culture of halophile bacterium and consequently remaining on the surface of the nanoparticles which were not completely removed during purification. Although the CFS of archaeal strain could produce silver nanoparticles (extracellular approach), but its EDX analysis (S3 Fig) showed various strong peaks related to P, Mg, and S suggesting the low level of its purity which was not selected for further analysis.

#### FTIR

**Fig 7:**
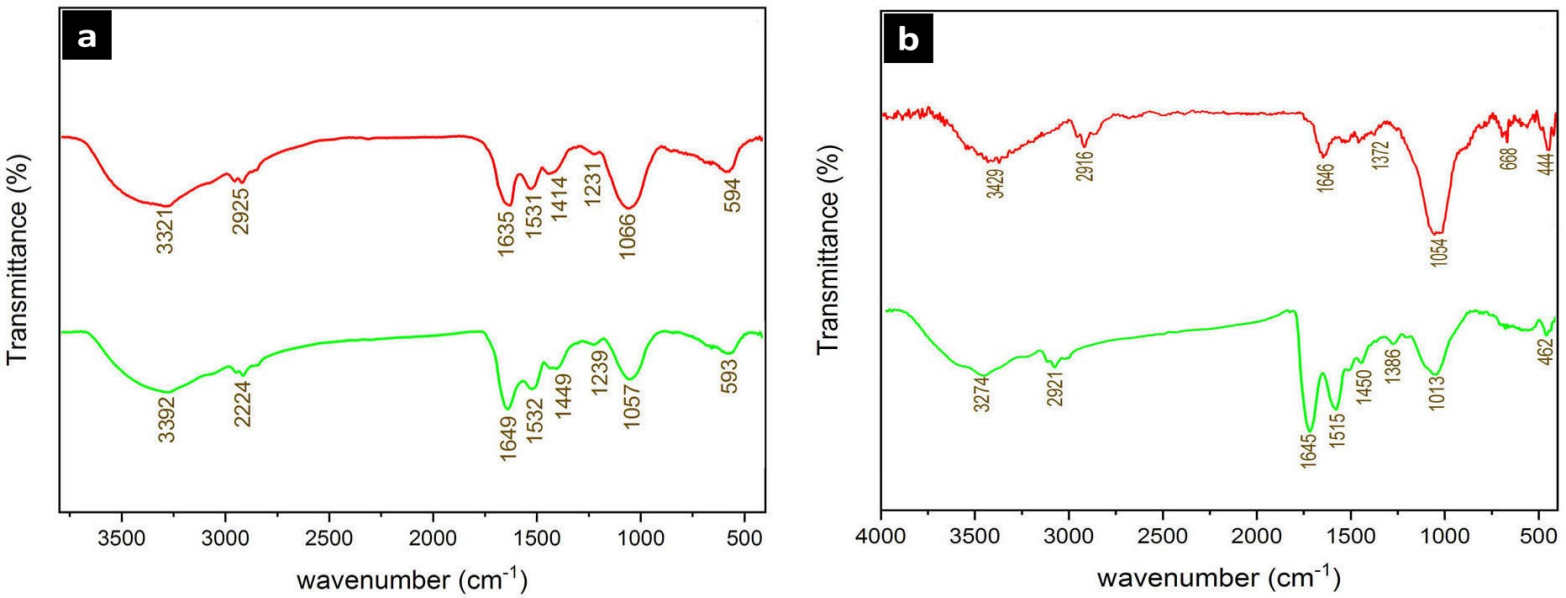
FT-IR spectrum of the silver (a) and selenium (b) nanoparticels. up: AgNPs-A and SeNPs-A. Down: AgNP-B and SeNP-B. AgNPs-A, silver nanoparticles synthesized by archaeal strain; SeNP-A, selenium nanoparticles synthesized by archaeal strain; AgNP-B, silver nanoparticles synthesized by bacterial strain; SeNP-B, selenium nanoparticles synthesized by bacterial strain.

The role of capping agents in this biogenesis approach which were present by some functional groups on the nanoparticles was confirmed by FTIR as presented in Fig 7A and 7B. The functional groups are responsible for the reduction and greater stability of nanoparticles. The FTIR spectrum showed characteristic absorption frequencies of the N-H stretching vibration amines and O-H group in phenols or alcohols at 3200–3600 cm-1, as well as C-H stretching and bending vibration in aldehydes at 2840–3000 cm-1. The C = O stretch of carbonyl groups in amino acid residues and peptides was observed at 1640–1690 cm-1 which have strong ability in binding to metal nanoparticles. The C = C stretch in aromatic compounds or N-H groups of secondary amines was also measured at 1400–1600 cm-1 which corresponds to heterocyclic compounds such as proteins. Furthermore, C-N stretching vibrations of aliphatic and aromatic amines were determined at 1020–1360 cm-1 with the bands at 675–1000 cm-1 being related to the C-H groups of aromatic compounds. All the above peaks appeared in both dried powder nanoparticles, indicating the capping groups surrounding the NPs. The FTIR spectrum of cell free supernatant (CFS) of the strain D1 applied in the extracellular synthesis of AgNP-B revealed the presence of peaks at 3401 and 3248 cm^−1^ that are corresponding to O-H stretching vibrations of alcohols and phenolic group of compounds along with the N-H stretching vibration amines. The peak observed at 1640 cm^−1^ represented the C = O stretch of carbonyl groups in amino acid residues and peptides. The C = C stretch in aromatic compounds or N-H groups of secondary amines was also measured at 1548 and 1417 cm^−1^ which corresponds to heterocyclic compounds such as proteins. Furthermore, the peak at 1327 cm^−1^ is representing the C-N stretching vibrations of aliphatic and aromatic amines with the bands at 1147 and 627 cm^−1^ being related to the C-O stretch and C-H groups of aromatic compounds (S4 Fig).

#### SEM/ TEM

**Fig 8:**
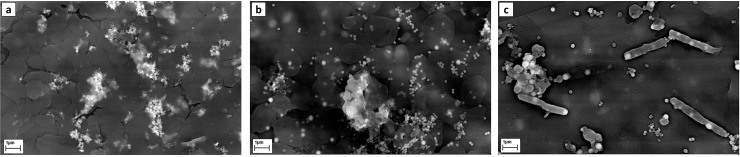
SEM images of the AgNP-A (a), SeNPs-A (b), and SeNPs-B (c) synthesized by the *Haloferax*, *Halogeometricum* and *Bacillus* strains, respectively.

**Fig 9:**
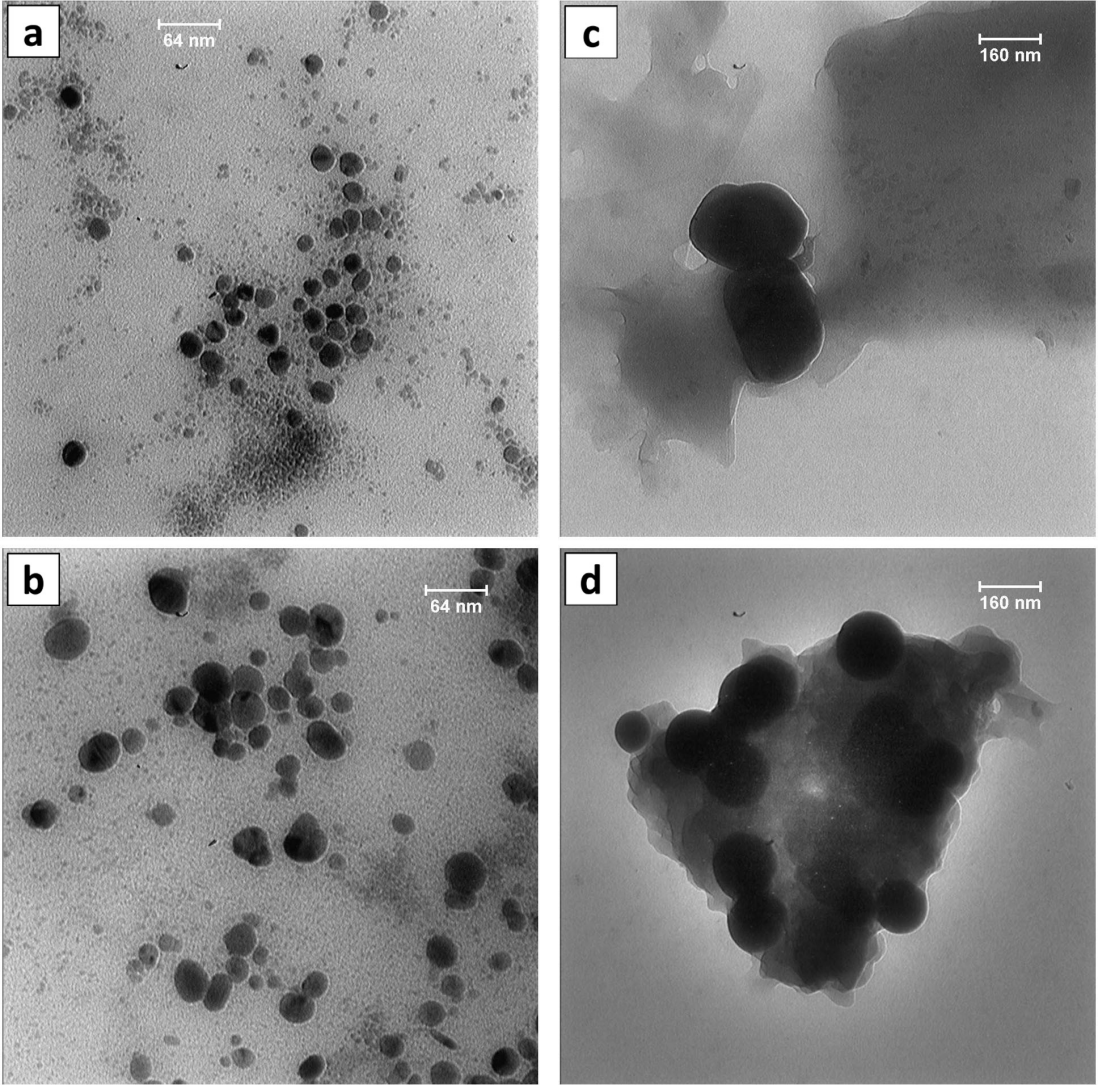
TEM images of the silver and selenium nanoparticles. a, AgNPs-A; b, AgNPs-B; c, SeNPs-A; d, SeNPs-B. AgNPs-A, silver nanoparticles synthesized by archaeal strain; AgNP-B, silver nanoparticles synthesized by bacterial strain; SeNP-A, selenium nanoparticles synthesized by archaeal strain; SeNP-B, selenium nanoparticles synthesized by bacterial strain.

Scanning electron microscopy analysis was conducted to represent the morphology and location of the intracellularly synthesized nanoparticles i.e., AgNP-A, SeNP-A, and SeNP-B. The pleomorphic archaeal cells for strains E106 and E118 were observed (Fig 8A and 8B), while silver and selenium nanoparticles were detected within or on the cells. AgNP-A and SeNP-A were mostly spherical in shape with both singular and aggregated particles. The bacillary shape of strain D45 with the intracellular and spherical selenium nanoparticles has been presented in Fig 7C. The chemical compositions of the nanoparticles were confirmed by EDX analysis. Concerning the extracellular synthesized AgNP-B, and other three intracellular nanoparticles (after purification), TEM analysis was performed to reveal the size and shape of the particles (Fig 9A–9D). Both of the archaeal and bacterial synthesized silver nanoparticles were predominantly spherical in shape with a few rod and disk like (especially in AgNPs-A) with the mean size of 26.34 and 22.10 nm respectively. Selenium nanoparticles produced by archaeal (SeNP-A) and bacterial (SeNP-B) cells were also spherical with the mean size of 111.6 and 141.7 nm, respectively (Fig 8C and 8D).

#### Antibacterial activity of AgNPS and SeNPs

**Fig 10:**
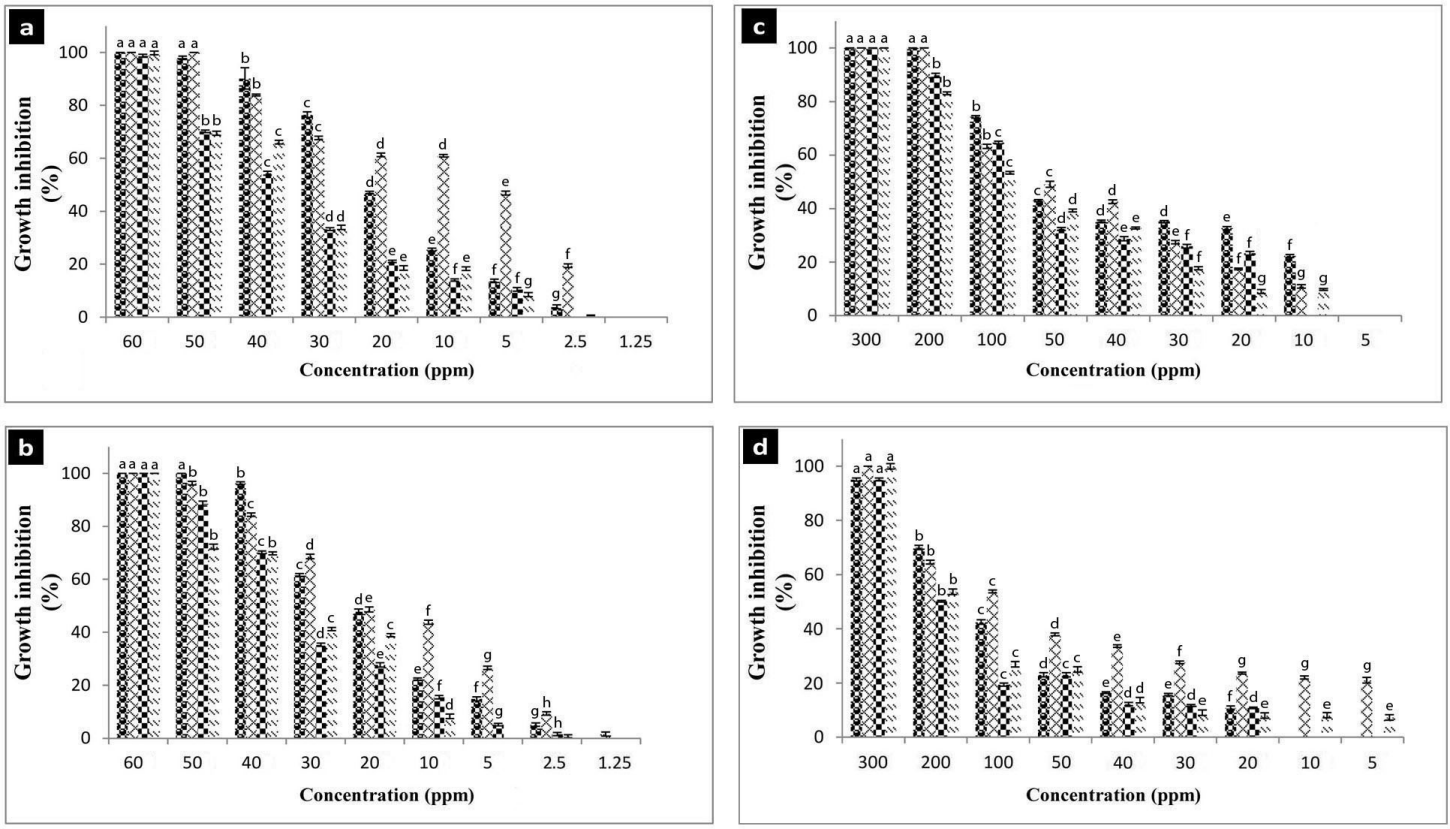
Antibacterial activity of the silver and selenium nanoparticles. a, AgNPs-A; b, AgNPs-B; c, SeNPs-A; d, SeNPs-B. AgNPs-A, silver nanoparticles synthesized by archaeal strain; AgNP-B, silver nanoparticles synthesized by bacterial strain; SeNP-A, selenium nanoparticles synthesized by archaeal strain; SeNP-B, selenium nanoparticles synthesized by bacterial strain. Microbial strains: sphere: *E*. *coli*; diamond: *S*. *aureus*; checker board: *B*. *subtilis*, dash: *P*. *aeruginosa*. Error bars indicate standard deviation for three replicates and different letters indicate significant difference between different concentrations (p<0.05).

Silver nanoparticles produced by both archaeal and bacterial strains (AgNP-A and AgNP-B) showed antibacterial activity against various bacterial pathogens. The MIC_50_ values of AgNP-A and Ag-NP-B against *E*. *coli*, *S*. *aureus*, *P*. *aeroginosa*, and *B*. *subtilis* were as: 20, 10, 40, 40 and 20, 20, 40, and 40 ppm, respectively. Regarding the antibacterial activity of selenium nanoparticles, the MIC_50_ of SeNP-A and Se-NP-B against *E*. *coli*, *S*. *aureus*, *P*. *aeroginosa*, and *B*. *subtilis* was 100, 50, 100, 100 and 200, 100, 200, and 200 ppm, respectively (Fig 10A–10D).

## Discussion

### Synthesis of nanoparticles

In this study, we used extreme and moderate halophiles isolated from a solar saltern for the green synthesis of silver and selenium nanoparticles. Extreme halophiles that could intracellularly synthesize silver (strain E106) and selenium (strain E108) nanoparticles belonged to the *Haloferax* and *Halogemetricum*, respectively. In a few reports regarding the potentials of haloarchaea for the synthesis of nanoparticles, Srivastava and co-workers found that the *Halococcous* strains could produce silver and selenium nanoparticles [24]. In another research, the ability of *Haloferax* sp. was presented for the green synthesis of silver nanoparticles [25]. In the current study, we extended this list by introducing the *Halogeometricum* strain as new halophilic archaeum with the potential to produce selenium nanoparticles. Consistent with our results, the abilities of *Halomonas* and *Bacillus* halophilic strains for the synthesis of gold and selenium nanoparticles were reported previously [26, 27]. The role of some reducates enzymes for the intracellulary of nanoparticles were presented in these studies. However, for the first time, the ability of *Halomanas* strain for the extracellular synthesis of silver nanoparticles was reported in the current study. The FTIR analysis of the CFS suggest that some protein compounds have role in the synthesis of extracellular AgNPs-B. The nanoparticles are capped and stabilized by these complex compounds which are secreted by strain D1 in the supernatant during its life cycle development. However, the reduction of colloidal silver ion by this supernatant extract was not observed at room temperature. Thus, an external source of energy was provided for biosynthesis of nanoparticles, to occur in the form of a hydrothermal autoclaving process (121° C and 15Ibs pressure). It is known that elevated temperature and pressure accelerate the synthesis of nanoparticles [28]. The slow and steady process of autoclaving wherein there is a gradual increase in the temperature from 0 to 121°C, steadily accelerated the synthesis of nanoparticles through the soar of free Ag^+^ ions from a lower energy state (electrons) into higher energy state thereby binding and embedding with functional groups of free secondary metabolites present in the reaction solution. In addition, autoclaving procedure completely reduces other microbial contamination potentially obtained during usage and transportation of nanoparticle synthesis. Desai et al. also successfully biosynthesized AgNPs by autoclaving *Streptomyces* sp. GUT21 supernatant [14].

### Characteristics of nanoparticles

The presence of silver and selenium nanoparticles was preliminary confirmed by Uv-Vis spectroscopy corresponding to typical plasmon resonance of oscillating and conducting electrons on the surface of nanoparticles. The absorption peak within 400–450 nm obtained by Uv-Vis spectroscopy represented the silver nanoparticles [29], while the peaks within 200–300 nm were related to selenium nanoparticles [30]; these are in accordance with the biosynthesized silver and selenium nanoparticles in this research. The development of nanocrystalline structures was evaluated based on x-ray diffraction. The XRD patterns of silver and selenium nanoparticles matched the JCPDS file no. 89–3722 and JCPDS file no. 06–0362, and they were recognized as face center cubic and hexagonal, respectively. Silver nanoparticles produced by archaeal strain (AgNP-A) showed an unassigned peak at 26.8°. This may be due to the organic compounds of the culture on the surface of the nanoparticles as approved in FTIR analysis [16, 31]. Some noises were observed in the backgrounds of SeNPs-A and SeNPs-B which might be related to bioactive compounds of archaeal and bacterial strain cultures in the intracellular biosynthesis approach [32]. With respect to the FWHM data of the maximum peaks, it was confirmed that the AgNP-B had a smaller size than AgNP-A. However, the silver nanocrystalline structure obtained by haloarchaeal strain (13.01nm) was smaller than that reported previously for AgNP produced by *Halococcus* strain (22 nm) [24]. The crystallite domain sizes of both selenium nanoparticles i.e., SeNP-A (30.63 nm) and SeNP-B (29.48 nm) were similar to a previous report for SeNPs by the halophilic strain, *Halococcus salifodinae* BK18 (28.27 nm) [33]. In comparison to the nanoparticles produced by non-halophiles like *Zooglea ramigera* (88.89 nm crystalline size) [34] and lemon leaf extract (60–80 nm crystalline size) [35], both SeNP-A and SeNP-B had a smaller crystallite domain size. Particle size distribution, revealed by DLS analysis, showed that archaeal and bacterial silver nanoparticles had similar distribution patterns, but the AgNP-B (mean size 52.6 nm) was relatively smaller than AgNP-A (82.42 nm). Regarding the selenium nanoparticle, SeNP-A was more uniform and smaller (118.3 nm) compared to SeNP-B (161.9 nm). The results were confirmed by their PDI value (0.27 vs 0.36). In comparison to some silver and selenium nanoparticles produced by non-halophiles such as *Actinotalea* (551.1 nm) [36], *Streptomyces* (44 nm) [14], and *Zooglea* (30–150 nm) [34] with PDI values of 0.5, 0.4, and 0.43, the current nanoparticles were more uniform.

The efficiency of nanoparticle production by these halophilic strains was relativity higher than that reported previously, such as 89% and 70% efficiency of selenium nanoparticle synthesis by *Pseudomonas putida* (92.4% in this study) and *Halococcous salifodinae* (84.8% in this study), respectively [17, 37]. The zeta potential indicates the degree of repulsion between adjacent, similarly charged particles in dispersion whose value can be related to the stability of colloidal dispersions [38]. Zeta potential analysis showed that the silver nanoparticles produced by halophilic bacteria and selenium nanoparticles produced by halophilic archaea were more stable (less zeta value) than those produced by their archaeal and bacterial counterparts, respectively. The zeta potentials of silver and selenium nanoparticles produced by other non-halophiles such as *Myxocoocus* [38], *Aspergillus* [18], and *Bacillus* [39] strains were reported as -25.2, -14, and -16.6 to -21.3, respectively. These data confirmed that the halophilic prokaryotes used in the current study produced the nanoparticles which are more stable than previously green synthesized silver and selenium nanoparticles.

The results of AFM analysis confirmed that most nanoparticles had a spherical and symmetrical morphology. The images revealed that silver and selenium produced by bacterial (mean size 24.8 nm) and archaeal strains (mean size 96.45 nm) had a smaller size compared to silver and selenium produced by the archaeal and bacterial strains, respectively. It was observed that larger particles formed due to aggregation of nanoparticles which might be attributed to the difficulty of precise cantilever tip attachment to the exact sample end during AFM set up, which might have increased discrepancy and variation in particles size [14]. FTIR analysis revealed the presence of C = O, C = C, N-H, C-N, and C-H groups of organic compounds suggesting the possible presence of amino acids, peptides, and aldehyde compounds on the surface of these nanoparticles. Similar to our results, the presence of these capping agents was reported on the silver and selenium nanoparticles produced by various microbial strains such as *Haloferax* [25] and *Lactobacillus* [20]. Low molecular peptides, glutathione (GSH), proteins such as metallothioneins and phytochelatins, enzymes such as oxidoreductases, NADH-dependent reductases, nitroreductases, and cysteine desulfhydrases have been shown to be responsible for nanocrystal formation in many microorganisms [24, 40]. Presence of these compounds in the supernatant and culture media of halophile strains has reduced potentials, increased the nanoparticle stability by their negative charges, and inhibited their agglomeration.

SEM analysis confirmed the nanoparticles in the microbial cells of strains E106, E118, and D54, approving the intracellular synthesis of nanoparticles, while their chemical compositions were confirmed by the EDX analysis. According to SEM images, selenium nanoparticles were uniformly shaped as spherical and polydisperse (especially in SeNPs-B). Very small and intracellular nano-spherical AgNPs-A were also achieved inside and outside the strain E106 cells which were aggregated in some places. The aggregation might be induced by the evaporation of solvent during sample preparation [14]. A smaller size of silver biosynthesized nanoparticles compared to their selenium counterpart was observed with the results being in accordance with AFM and TEM analysis. TEM images also confirmed the uniform, dispersed, and most spherical structures of silver and selenium nanoparticles. The calculated average sizes of AgNPs-B (22.1 nm) and SeNPs- A (111.6 nm) was smaller than the AgNPs-A (26.34 nm) and SeNPs-B (141.6 nm). The size of AgNPs and SeNPs produced by previously reported halophilic *Halococcus salifodinae* BK3 [24], *Halococcus salifodinae* BK18 [33], and non-halophilic *Klebsiella pneumonia* [30] had the mean size of 50 and 129, and 245 nm, respectively, suggesting the smaller size of the nanoparticles produced in this research.

The antibacterial study revealed that the silver nanoparticles were more toxic to the bacterial pathogens than selenium nanoparticles were. In comparison to the previously reported antibacterial activity of biogenic nanoparticles, the silver and selenium nanoparticles produced in this study showed a moderate antibacterial activity. This can be related to their extreme nature where their capping agents cannot enhance the biological activity of the nanoparticles under non-extreme conditions.

## Conclusion

The halophilic archaea and bacteria can produce silver and selenium nanoparticles. The AgNPs and SeNPs produced by halophilic strain showed excellent properties regarding their size, size uniformity, stability, and purity in comparison to the various previously reported nanoparticles produced by (none) halophilic prokaryotes. Silver nanoparticles produced by halophilic bacteria (*Halomonas*) had more notable physicochemical properties compared to the silver nanoparticles produced by the archaeal strain (*Halogometricum*). Regarding the selenium nanoparticles, archaeal-based nanoparticles (*Haloferax*) showed better properties like the smaller size, higher stability, and lower polydispersity index compared to the bacterial (*Bacillus*) ones. The results of this study suggested that the (extremely) halophilic prokaryotes have great potentials for the green synthesis of the nanoparticles.

## Supporting information

S1 Fig The resistance of extreme (vertical dash) and moderate (horizontal dash) halophiles to the silver nitrate (a) and sodium selenite (b) salts.
(TIF)

S2 Fig The liquid cultures showed the production of AgNPs-A (a), SeNPs-A (b), and SeNPs-B (c) by different strains.
(TIF)

S3 Fig Energy dispersive X-ray (EDX) spectrum of the silver nanoparticels produced by archaeal cell free supernatant. (TIF)

S4 Fig FT-IR spectrum of the strain D1 cell free supernatant. (TIF)
