# Supplementary figures and images for: Exploring the potentials of halophilic prokaryotes from a solar saltern for synthesizing nanoparticles: The case of silver and selenium
Source: PLoS One. 2020 Mar 4;15(3):e0229886. doi: 10.1371/journal.pone.0229886 (PMC7055902; doi:10.1371/journal.pone.0229886)

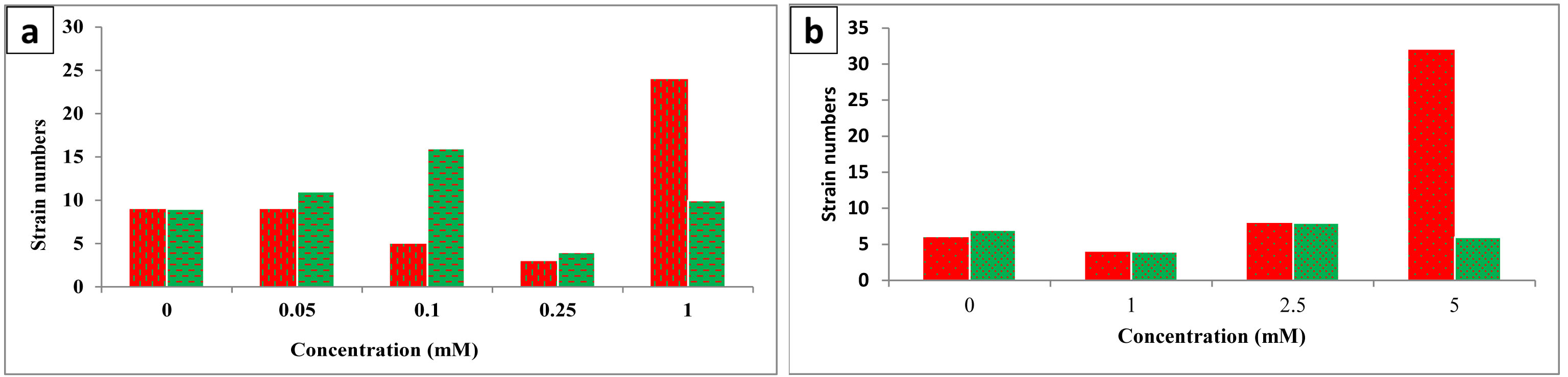

Supplement: S1 Fig — The resistance of extreme (vertical dash) and moderate (horizontal dash) halophiles to the silver nitrate (a) and sodium selenite (b) salts. (TIF) [file pone.0229886.s001.tif]

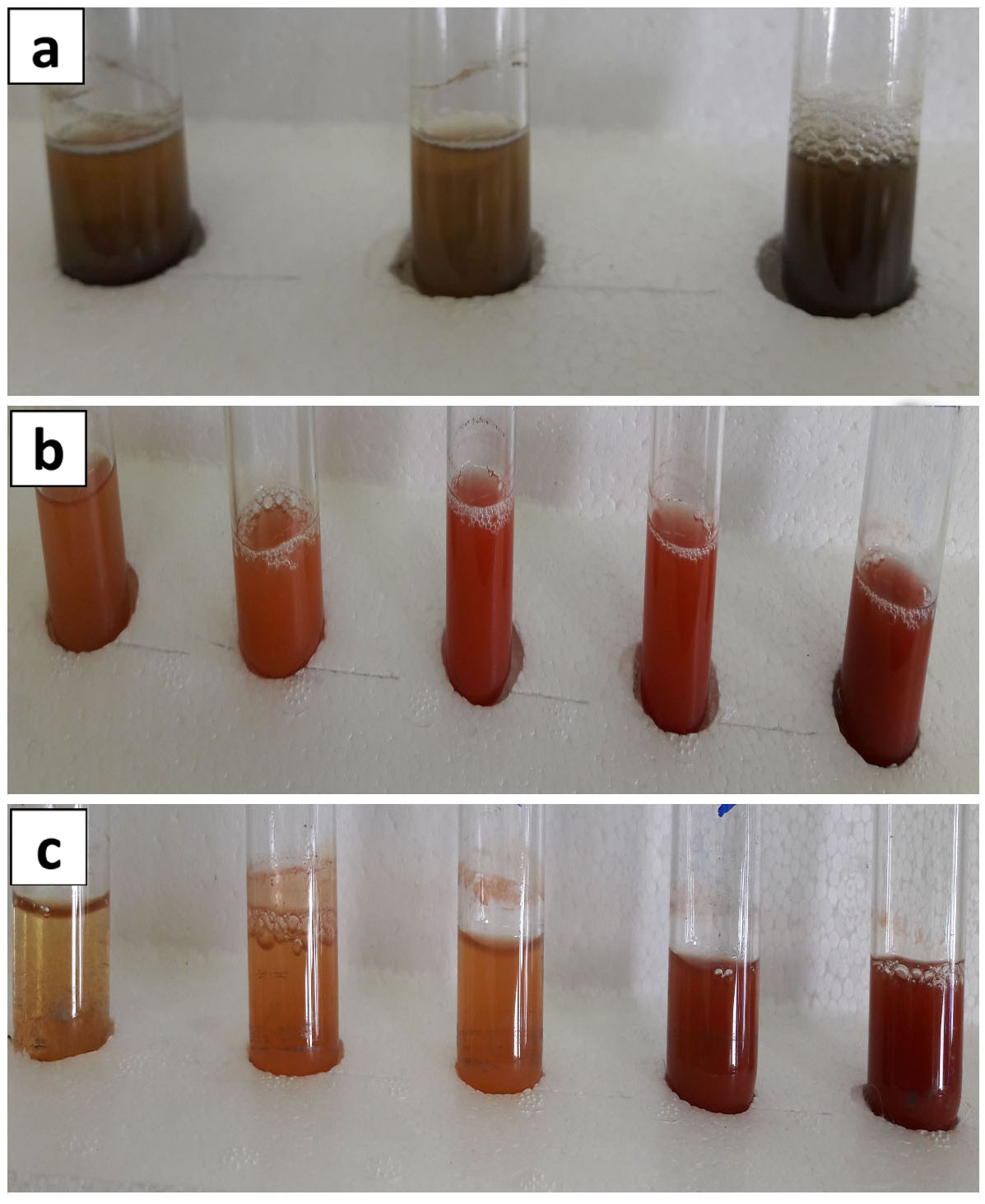

Supplement: S2 Fig — The liquid cultures showed the production of AgNPs-A (a), SeNPs-A (b), and SeNPs-B (c) by different strains. (TIF) [file pone.0229886.s002.tif]

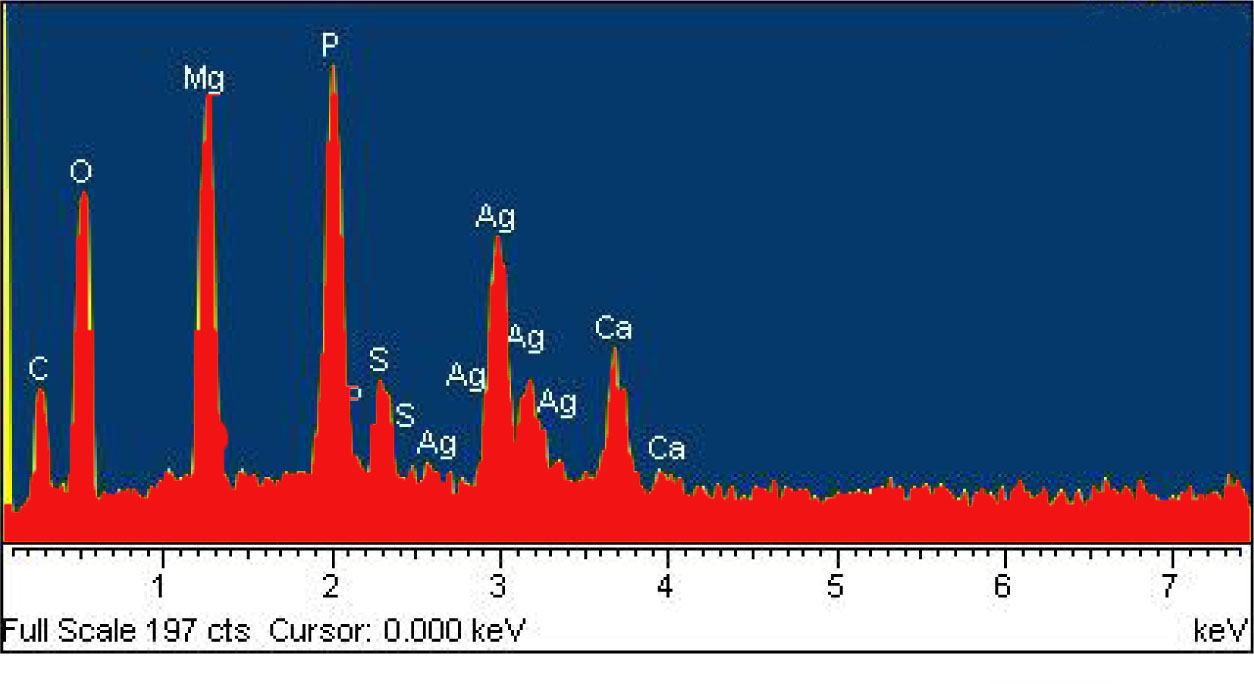

Supplement: S3 Fig — (TIF) [file pone.0229886.s003.tif]

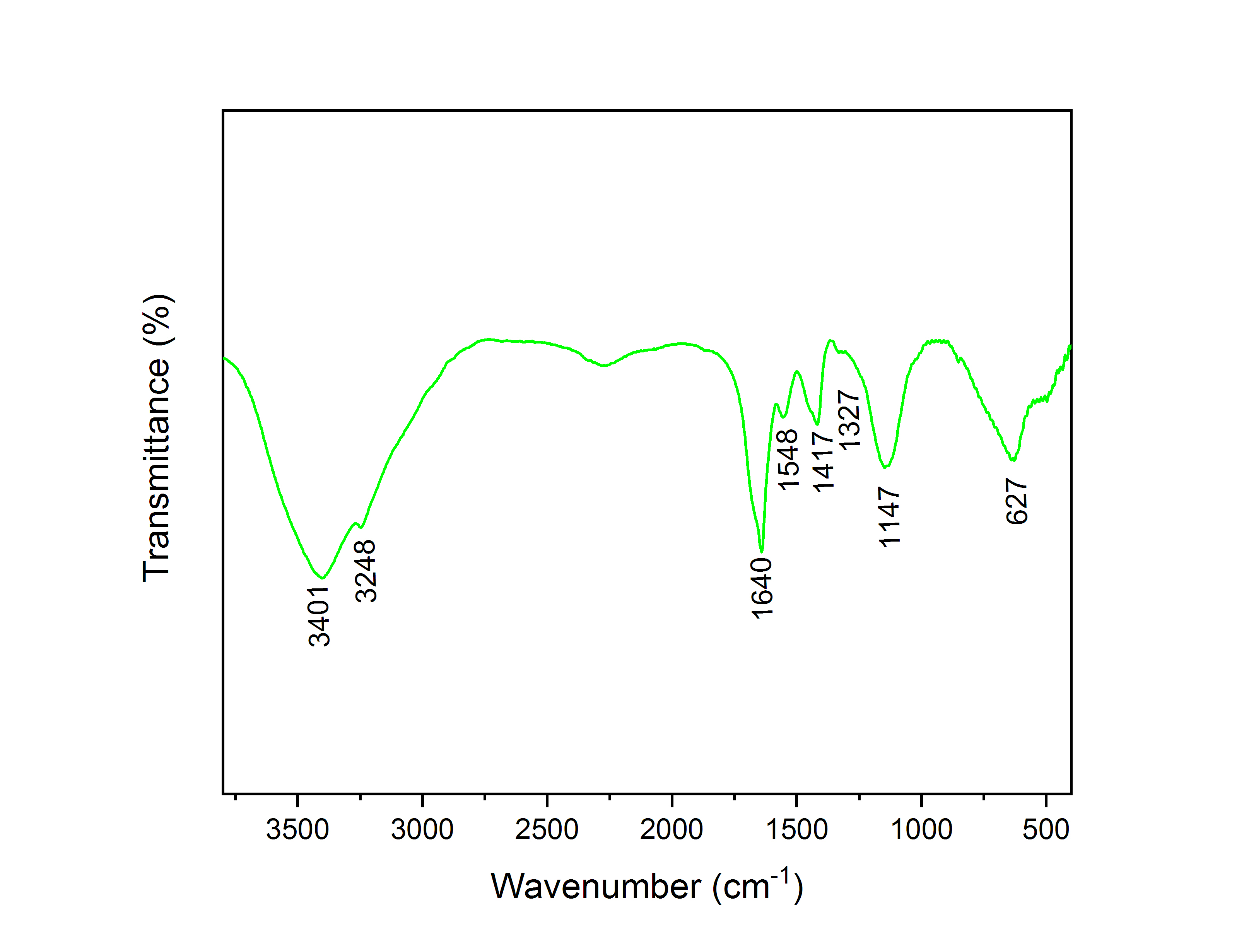

Supplement: S4 Fig — (TIF) [file pone.0229886.s004.tif]
